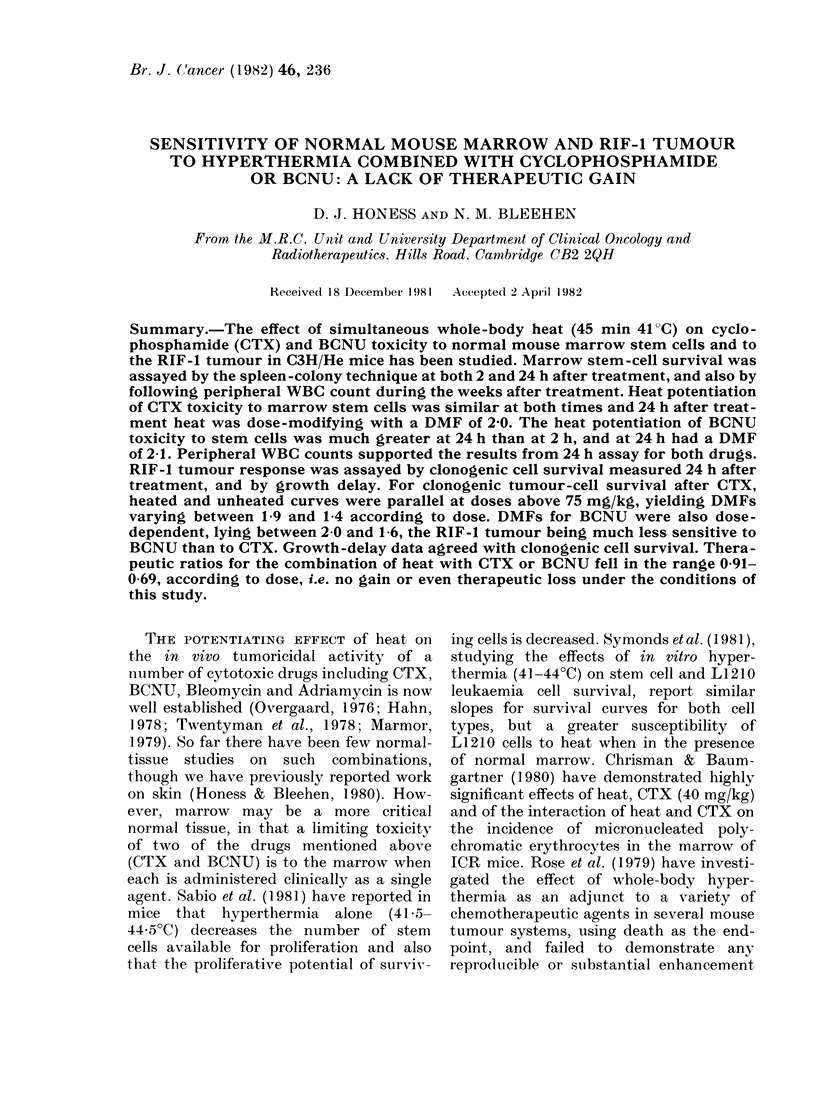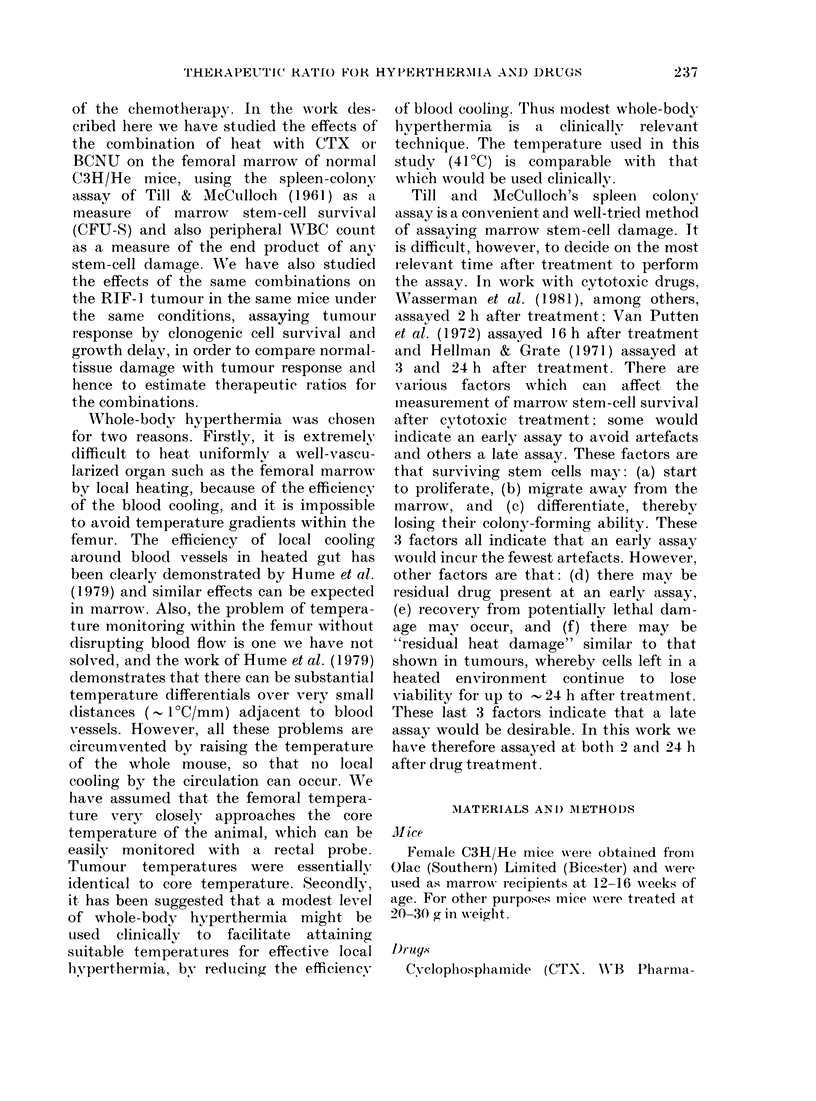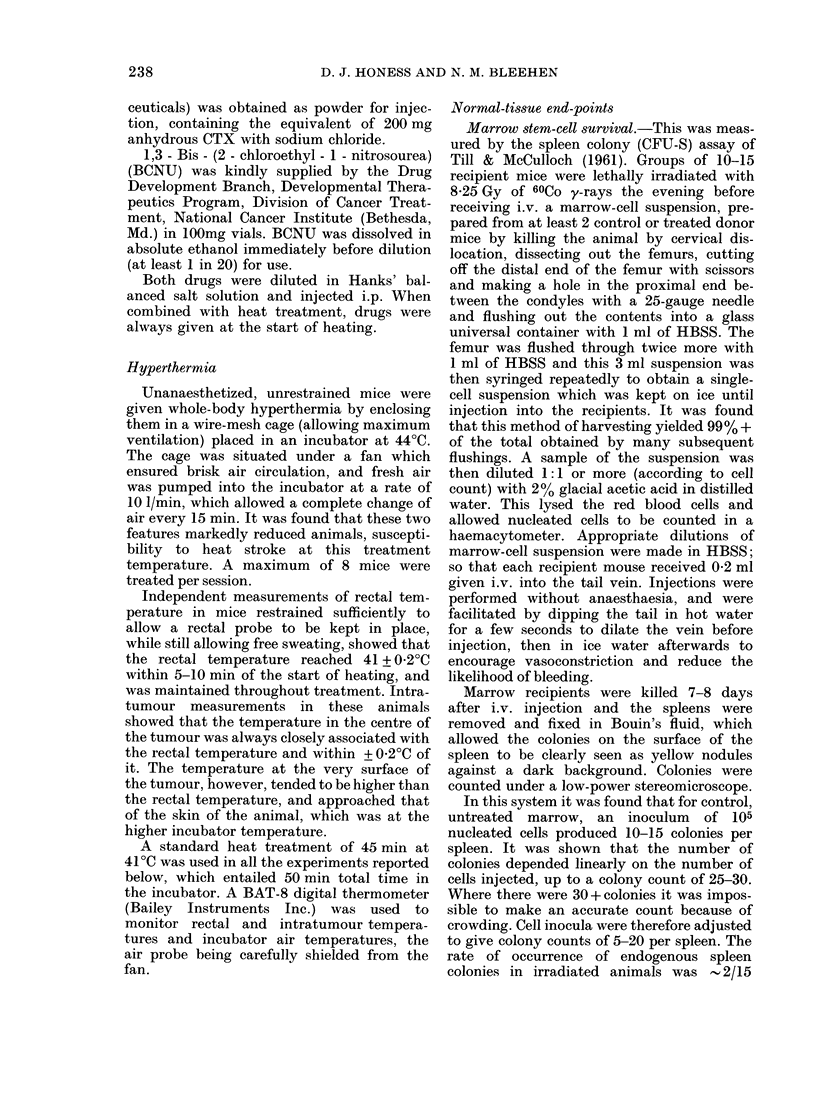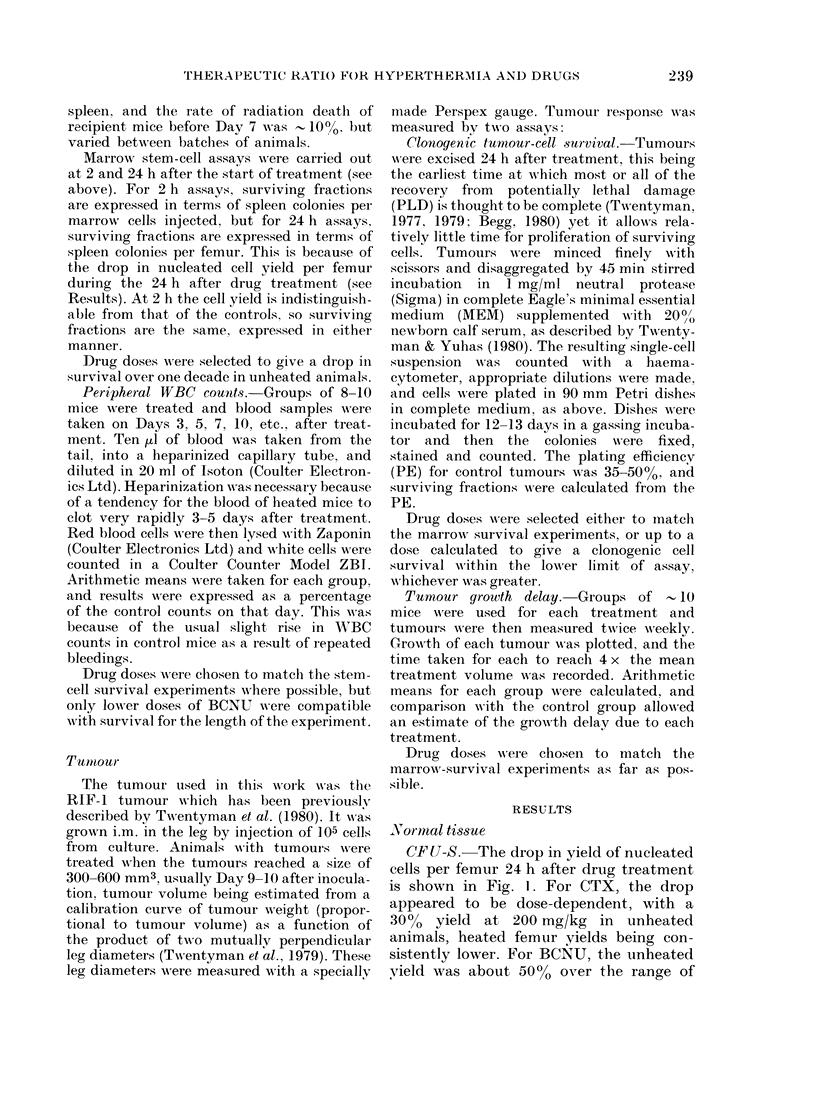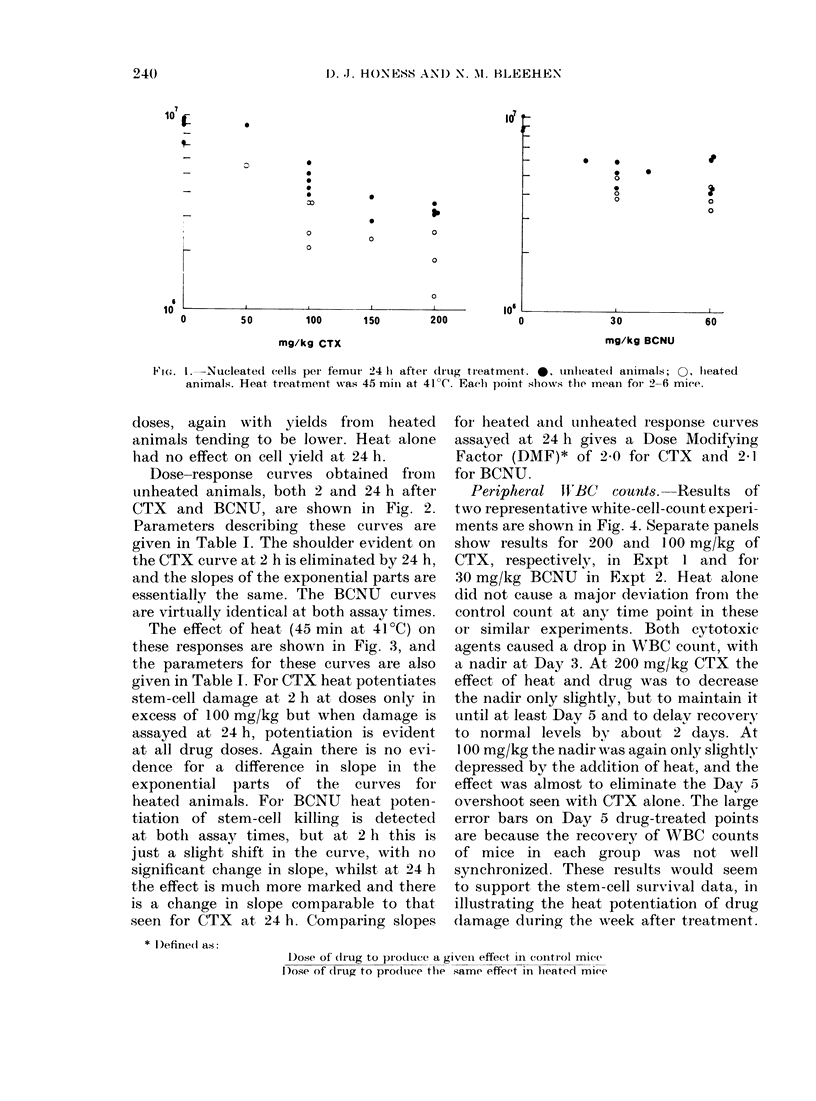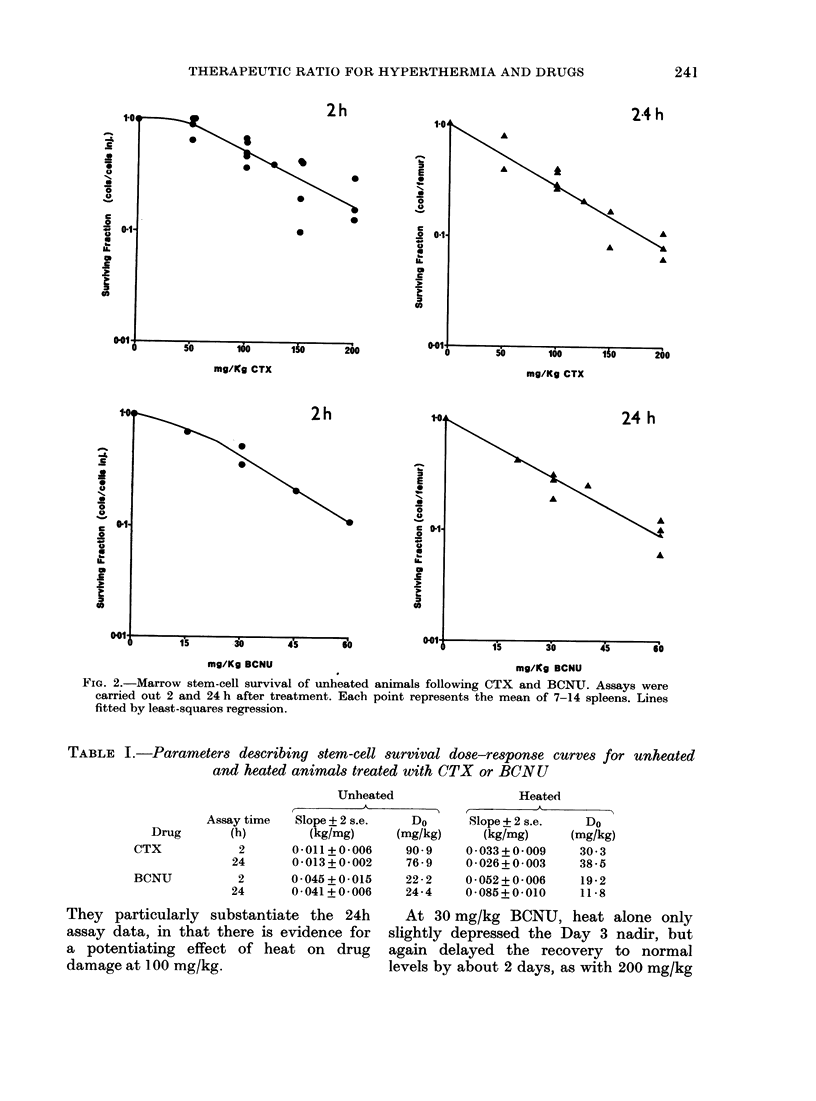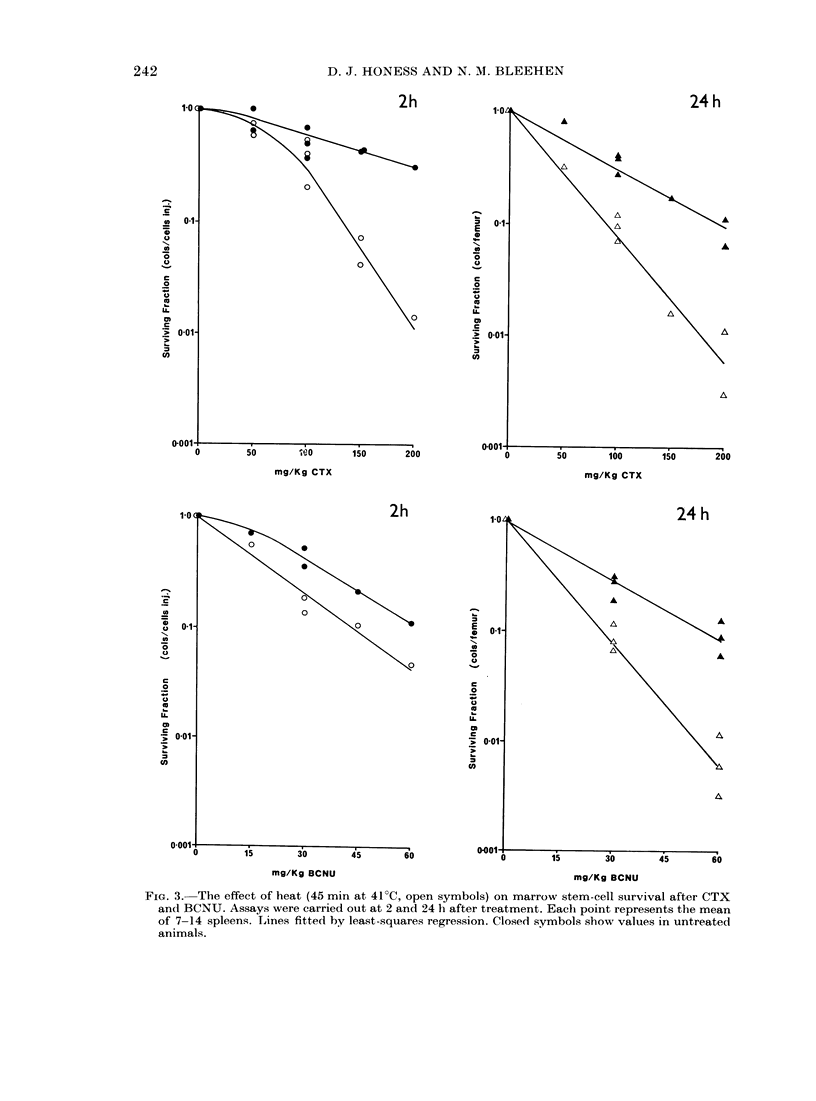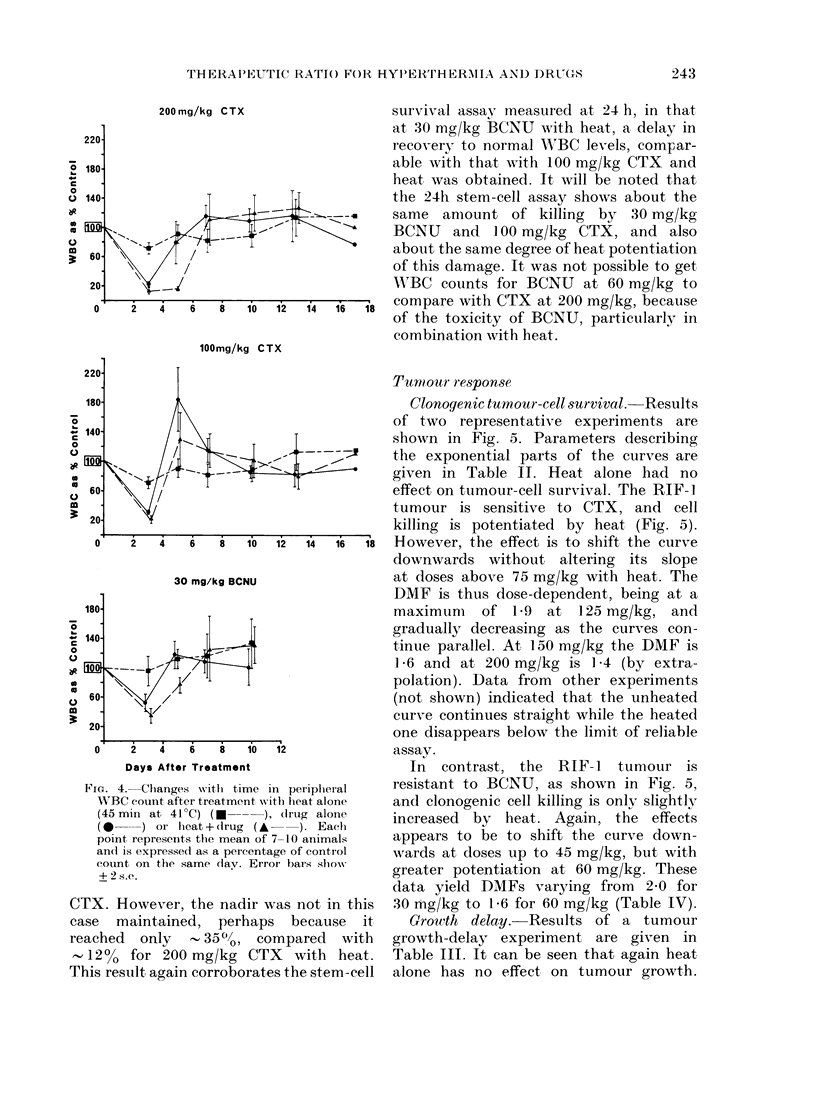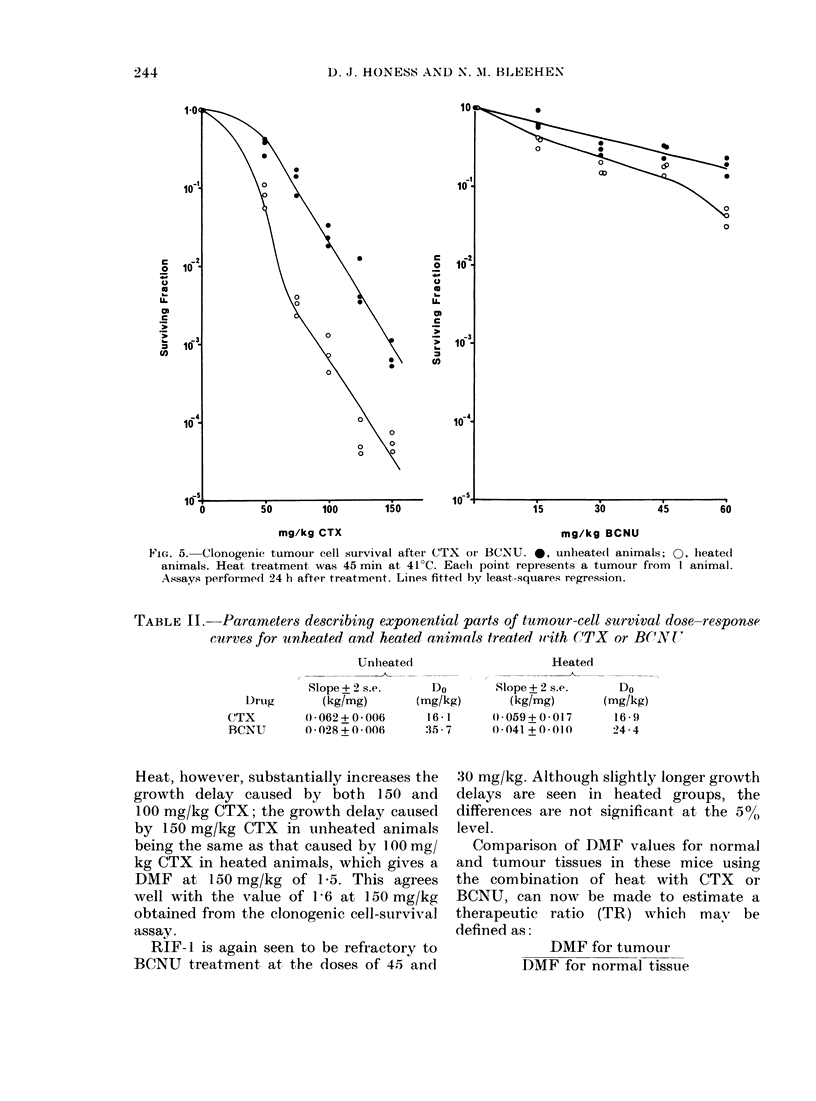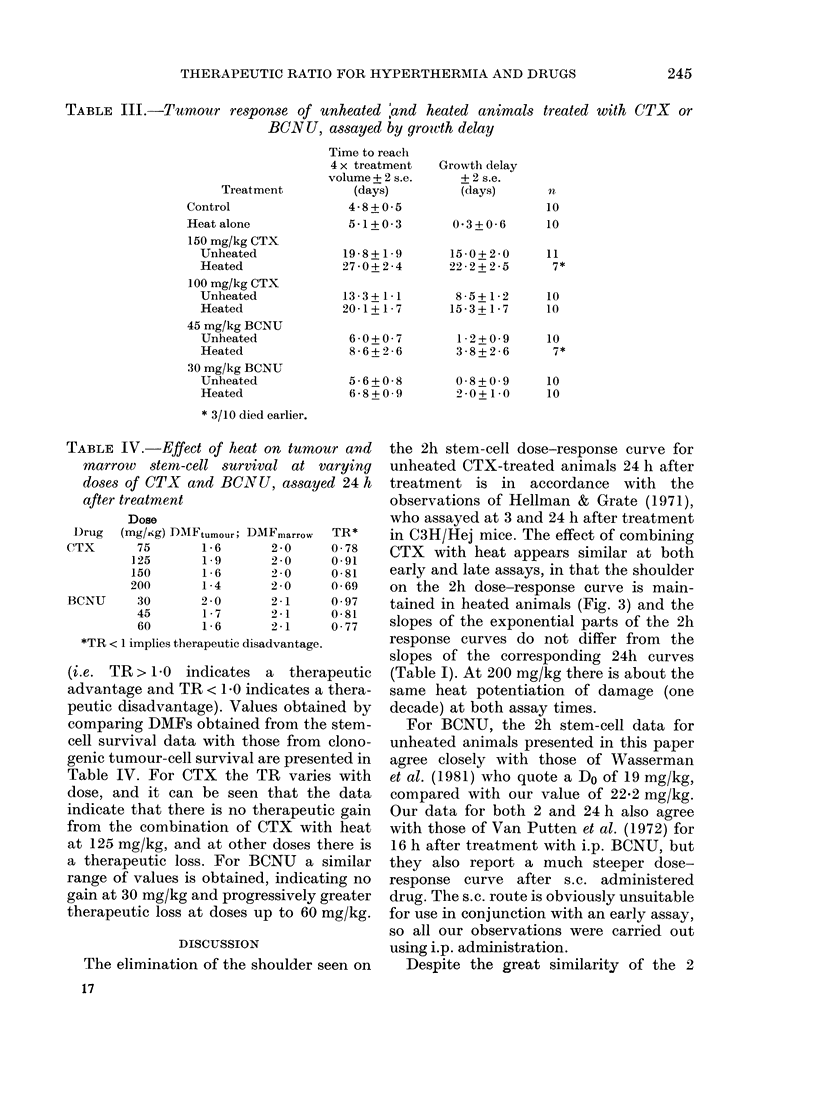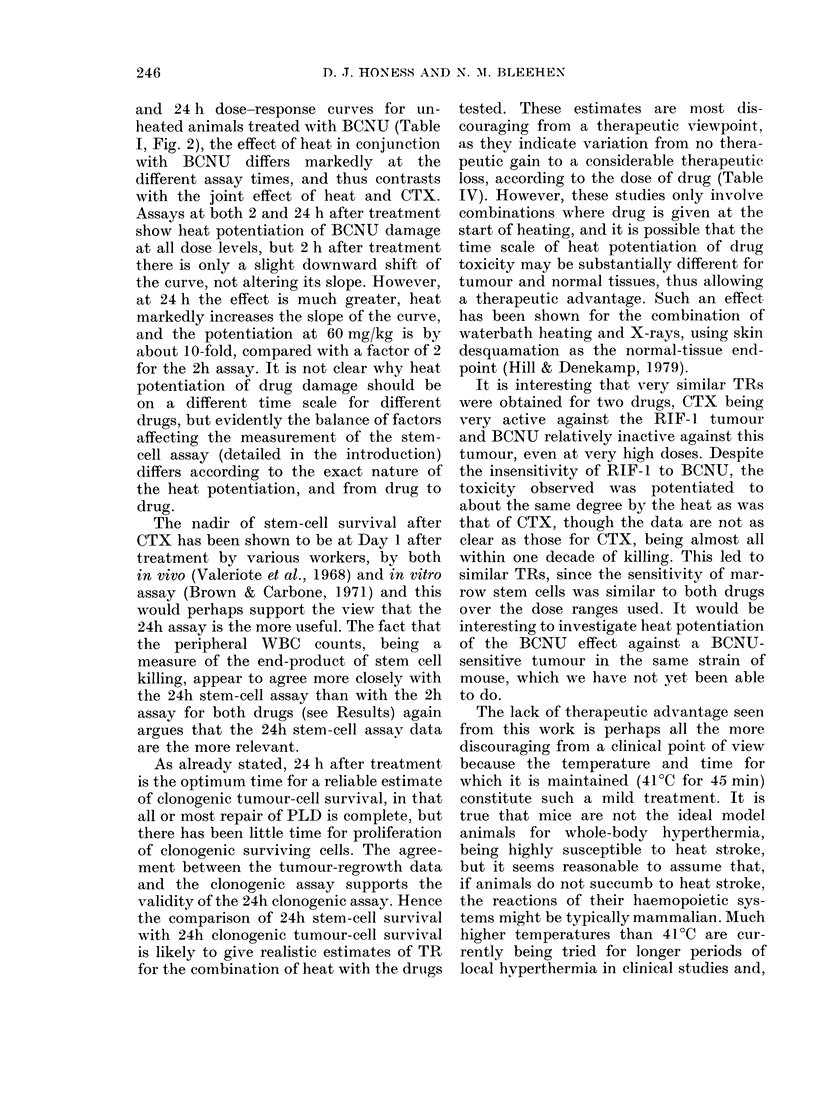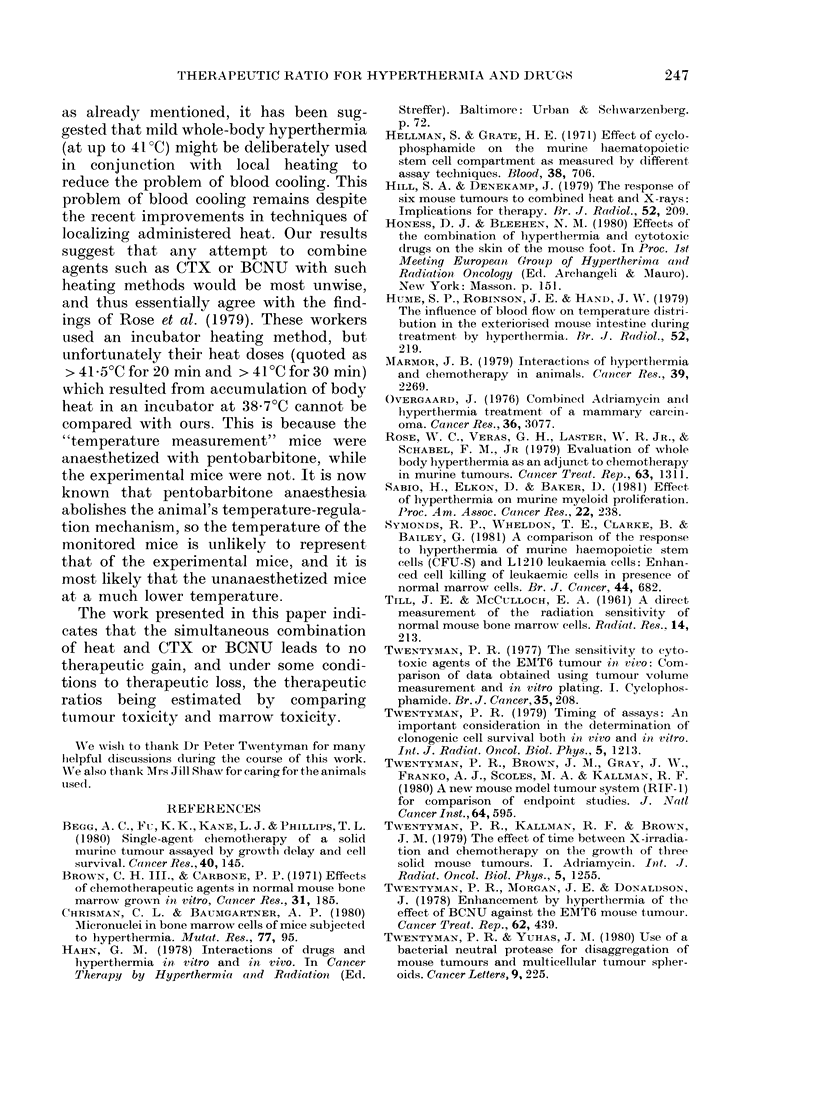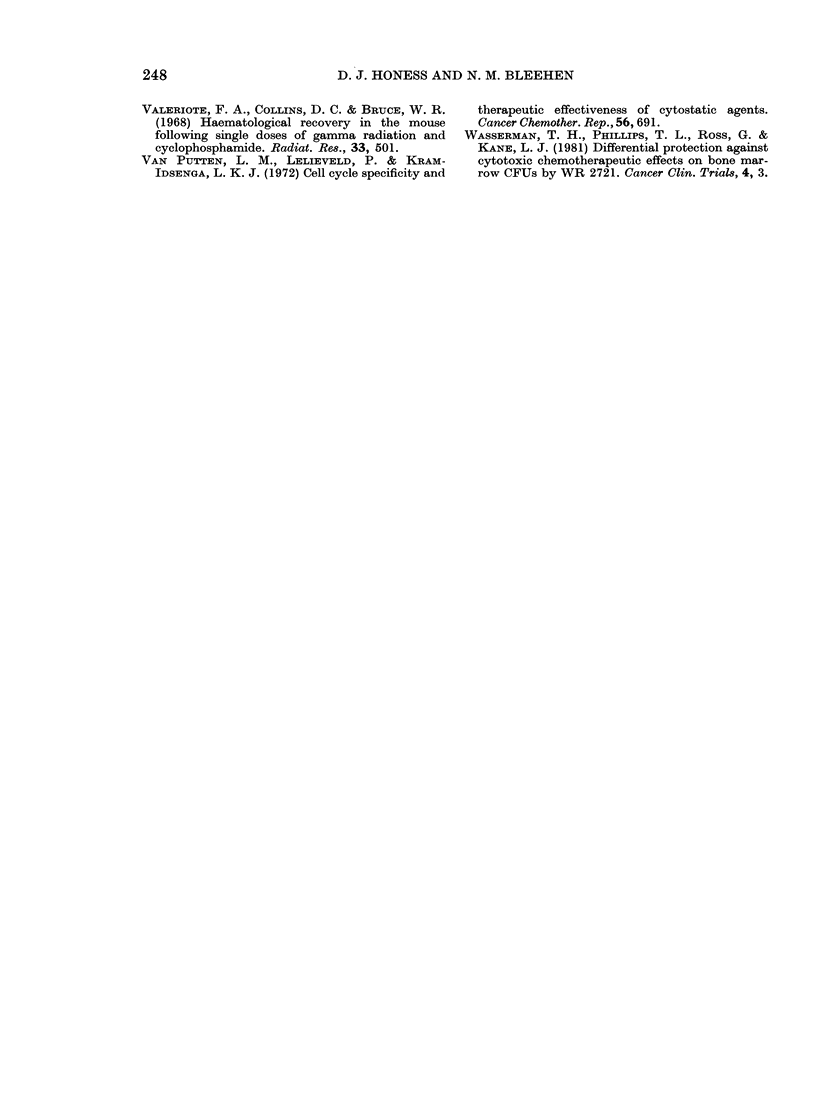# Sensitivity of normal mouse marrow and RIF-1 tumour to hyperthermia combined with cyclophosphamide or BCNU: a lack of therapeutic gain.

**DOI:** 10.1038/bjc.1982.189

**Published:** 1982-08

**Authors:** D. J. Honess, N. M. Bleehen

## Abstract

The effect of simultaneous whole-body heat (45 min 41 degrees C) on cyclophosphamide (CTX) and BCNU toxicity to normal mouse marrow stem cells and to the RIF-1 tumour in C3H/He mice has been studied. Marrow stem-cell survival was assayed by the spleen-colony technique at both 2 and 24 h after treatment, and also by following peripheral WBC count during the weeks after treatment. Heat potentiation of CTX toxicity to marrow stem cells was similar at both times and 24 h after treatment heat was dose-modifying with a DMF of 2.0. The heat potentiation of BCNU toxicity to stem cells was much greater at 24 h than at 2 h, and at 24 h had a DMF of 2.1. Peripheral WBC counts supported the results from 24 h assay for both drugs. RIF-1 tumour response was assayed by clonogenic cell survival measured 24 h after treatment, and by growth delay. For clonogenic tumour-cell survival after CTX, heated and unheated curves were parallel at doses above 75 mg/kg, yielding DMFs varying between 1.9 and 1.4 according to dose. DMFs for BCNU were also dose-dependent, lying between 2.0 and 1.6, the RIF-1 tumour being much less sensitive to BCNU than to CTX. Growth-delay data agreed with clonogenic cell survival. Therapeutic ratios for the combination of heat with CTX or BCNU fell in the range 0.91--0.69, according to dose, i.e. no gain or even therapeutic loss under the conditions of this study.


*Br. J. Cancer* (1982) **46**, 236

### SENSITIVITY OF NORMAL MOUSE MARROW AND RIF-1 TUMOUR TO HYPERTHERMIA COMBINED WITH CYCLOPHOSPHAMIDE OR BCNU: A LACK OF THERAPEUTIC GAIN

D. J. HONESS AND N. M. BLEEHEN

*From the M.R.C. Unit and University Department of Clinical Oncology and Radiotherapeutics, Hills Road, Cambridge CB2 2QH*

Received 18 December 1981 Accepted 2 April 1982

**Summary.—The effect of simultaneous whole-body heat (45 min 41°C) on cyclophosphamide (CTX) and BCNU toxicity to normal mouse marrow stem cells and to the RIF-1 tumour in C3H/He mice has been studied. Marrow stem-cell survival was assayed by the spleen-colony technique at both 2 and 24 h after treatment, and also by following peripheral WBC count during the weeks after treatment. Heat potentiation of CTX toxicity to marrow stem cells was similar at both times and 24 h after treatment heat was dose-modifying with a DMF of 2·0. The heat potentiation of BCNU toxicity to stem cells was much greater at 24 h than at 2 h, and at 24 h had a DMF of 2·1. Peripheral WBC counts supported the results from 24 h assay for both drugs. RIF-1 tumour response was assayed by clonogenic cell survival measured 24 h after treatment, and by growth delay. For clonogenic tumour-cell survival after CTX, heated and unheated curves were parallel at doses above 75 mg/kg, yielding DMFs varying between 1·9 and 1·4 according to dose. DMFs for BCNU were also dose-dependent, lying between 2·0 and 1·6, the RIF-1 tumour being much less sensitive to BCNU than to CTX. Growth-delay data agreed with clonogenic cell survival. Therapeutic ratios for the combination of heat with CTX or BCNU fell in the range 0·91–0·69, according to dose, *i.e.* no gain or even therapeutic loss under the conditions of this study.**

THE POTENTIATING EFFECT of heat on the *in vivo* tumoricidal activity of a number of cytotoxic drugs including CTX, BCNU, Bleomycin and Adriamycin is now well established (Overgaard, 1976; Hahn, 1978; Twentyman *et al.*, 1978; Marmor, 1979). So far there have been few normal-tissue studies on such combinations, though we have previously reported work on skin (Honess & Bleehen, 1980). However, marrow may be a more critical normal tissue, in that a limiting toxicity of two of the drugs mentioned above (CTX and BCNU) is to the marrow when each is administered clinically as a single agent. Sabio *et al.* (1981) have reported in mice that hyperthermia alone (41·5–44·5°C) decreases the number of stem cells available for proliferation and also that the proliferative potential of surviving cells is decreased. Symonds *et al.* (1981), studying the effects of *in vitro* hyperthermia (41–44°C) on stem cell and L1210 leukaemia cell survival, report similar slopes for survival curves for both cell types, but a greater susceptibility of L1210 cells to heat when in the presence of normal marrow. Chrisman & Baumgartner (1980) have demonstrated highly significant effects of heat, CTX (40 mg/kg) and of the interaction of heat and CTX on the incidence of micronucleated polychromatic erythrocytes in the marrow of ICR mice. Rose *et al.* (1979) have investigated the effect of whole-body hyperthermia as an adjunct to a variety of chemotherapeutic agents in several mouse tumour systems, using death as the end-point, and failed to demonstrate any reproducible or substantial enhancement of the chemotherapy. In the work described here we have studied the effects of the combination of heat with CTX or BCNU on the femoral marrow of normal C3H/He mice, using the spleen-colony assay of Till & McCulloch (1961) as a measure of marrow stem-cell survival (CFU-S) and also peripheral WBC count as a measure of the end product of any stem-cell damage. We have also studied the effects of the same combinations on the RIF-1 tumour in the same mice under the same conditions, assaying tumour response by clonogenic cell survival and growth delay, in order to compare normal-tissue damage with tumour response and hence to estimate therapeutic ratios for the combinations.

Whole-body hyperthermia was chosen for two reasons. Firstly, it is extremely difficult to heat uniformly a well-vascularized organ such as the femoral marrow by local heating, because of the efficiency of the blood cooling, and it is impossible to avoid temperature gradients within the femur. The efficiency of local cooling around blood vessels in heated gut has been clearly demonstrated by Hume *et al.* (1979) and similar effects can be expected in marrow. Also, the problem of temperature monitoring within the femur without disrupting blood flow is one we have not solved, and the work of Hume *et al.* (1979) demonstrates that there can be substantial temperature differentials over very small distances ($\sim 1°C/mm$) adjacent to blood vessels. However, all these problems are circumvented by raising the temperature of the whole mouse, so that no local cooling by the circulation can occur. We have assumed that the femoral temperature very closely approaches the core temperature of the animal, which can be easily monitored with a rectal probe. Tumour temperatures were essentially identical to core temperature. Secondly, it has been suggested that a modest level of whole-body hyperthermia might be used clinically to facilitate attaining suitable temperatures for effective local hyperthermia, by reducing the efficiency of blood cooling. Thus modest whole-body hyperthermia is a clinically relevant technique. The temperature used in this study (41°C) is comparable with that which would be used clinically.

Till and McCulloch's spleen colony assay is a convenient and well-tried method of assaying marrow stem-cell damage. It is difficult, however, to decide on the most relevant time after treatment to perform the assay. In work with cytotoxic drugs, Wasserman *et al.* (1981), among others, assayed 2 h after treatment; Van Putten *et al.* (1972) assayed 16 h after treatment and Hellman & Grate (1971) assayed at 3 and 24 h after treatment. There are various factors which can affect the measurement of marrow stem-cell survival after cytotoxic treatment; some would indicate an early assay to avoid artefacts and others a late assay. These factors are that surviving stem cells may: (a) start to proliferate, (b) migrate away from the marrow, and (c) differentiate, thereby losing their colony-forming ability. These 3 factors all indicate that an early assay would incur the fewest artefacts. However, other factors are that: (d) there may be residual drug present at an early assay, (e) recovery from potentially lethal damage may occur, and (f) there may be "residual heat damage" similar to that shown in tumours, whereby cells left in a heated environment continue to lose viability for up to $\sim 24$ h after treatment. These last 3 factors indicate that a late assay would be desirable. In this work we have therefore assayed at both 2 and 24 h after drug treatment.

### MATERIALS AND METHODS

### *Mice*

Female C3H/He mice were obtained from Olac (Southern) Limited (Bicester) and were used as marrow recipients at 12–16 weeks of age. For other purposes mice were treated at 20–30 g in weight.

### *Drugs*

Cyclophosphamide (CTX, WB Pharmaceuticals) was obtained as powder for injection, containing the equivalent of 200 mg anhydrous CTX with sodium chloride.

1,3 - Bis - (2 - chloroethyl - 1 - nitrosourea) (BCNU) was kindly supplied by the Drug Development Branch, Developmental Therapeutics Program, Division of Cancer Treatment, National Cancer Institute (Bethesda, Md.) in 100mg vials. BCNU was dissolved in absolute ethanol immediately before dilution (at least 1 in 20) for use.

Both drugs were diluted in Hanks' balanced salt solution and injected i.p. When combined with heat treatment, drugs were always given at the start of heating.

### *Hyperthermia*

Unanaesthetized, unrestrained mice were given whole-body hyperthermia by enclosing them in a wire-mesh cage (allowing maximum ventilation) placed in an incubator at 44°C. The cage was situated under a fan which ensured brisk air circulation, and fresh air was pumped into the incubator at a rate of 10 l/min, which allowed a complete change of air every 15 min. It was found that these two features markedly reduced animals, susceptibility to heat stroke at this treatment temperature. A maximum of 8 mice were treated per session.

Independent measurements of rectal temperature in mice restrained sufficiently to allow a rectal probe to be kept in place, while still allowing free sweating, showed that the rectal temperature reached $41 \pm 0{\cdot}2$°C within 5–10 min of the start of heating, and was maintained throughout treatment. Intratumour measurements in these animals showed that the temperature in the centre of the tumour was always closely associated with the rectal temperature and within $\pm 0{\cdot}2$°C of it. The temperature at the very surface of the tumour, however, tended to be higher than the rectal temperature, and approached that of the skin of the animal, which was at the higher incubator temperature.

A standard heat treatment of 45 min at 41°C was used in all the experiments reported below, which entailed 50 min total time in the incubator. A BAT-8 digital thermometer (Bailey Instruments Inc.) was used to monitor rectal and intratumour temperatures and incubator air temperatures, the air probe being carefully shielded from the fan.

### *Normal-tissue end-points*

*Marrow stem-cell survival.*—This was measured by the spleen colony (CFU-S) assay of Till & McCulloch (1961). Groups of 10–15 recipient mice were lethally irradiated with 8·25 Gy of $^{60}$Co $\gamma$-rays the evening before receiving i.v. a marrow-cell suspension, prepared from at least 2 control or treated donor mice by killing the animal by cervical dislocation, dissecting out the femurs, cutting off the distal end of the femur with scissors and making a hole in the proximal end between the condyles with a 25-gauge needle and flushing out the contents into a glass universal container with 1 ml of HBSS. The femur was flushed through twice more with 1 ml of HBSS and this 3 ml suspension was then syringed repeatedly to obtain a single-cell suspension which was kept on ice until injection into the recipients. It was found that this method of harvesting yielded 99%+ of the total obtained by many subsequent flushings. A sample of the suspension was then diluted 1:1 or more (according to cell count) with 2% glacial acetic acid in distilled water. This lysed the red blood cells and allowed nucleated cells to be counted in a haemacytometer. Appropriate dilutions of marrow-cell suspension were made in HBSS; so that each recipient mouse received 0·2 ml given i.v. into the tail vein. Injections were performed without anaesthaesia, and were facilitated by dipping the tail in hot water for a few seconds to dilate the vein before injection, then in ice water afterwards to encourage vasoconstriction and reduce the likelihood of bleeding.

Marrow recipients were killed 7–8 days after i.v. injection and the spleens were removed and fixed in Bouin's fluid, which allowed the colonies on the surface of the spleen to be clearly seen as yellow nodules against a dark background. Colonies were counted under a low-power stereomicroscope.

In this system it was found that for control, untreated marrow, an inoculum of $10^5$ nucleated cells produced 10–15 colonies per spleen. It was shown that the number of colonies depended linearly on the number of cells injected, up to a colony count of 25–30. Where there were 30+ colonies it was impossible to make an accurate count because of crowding. Cell inocula were therefore adjusted to give colony counts of 5–20 per spleen. The rate of occurrence of endogenous spleen colonies in irradiated animals was $\sim$2/15

spleen, and the rate of radiation death of recipient mice before Day 7 was ~10%, but varied between batches of animals.

Marrow stem-cell assays were carried out at 2 and 24 h after the start of treatment (see above). For 2 h assays, surviving fractions are expressed in terms of spleen colonies per marrow cells injected, but for 24 h assays, surviving fractions are expressed in terms of spleen colonies per femur. This is because of the drop in nucleated cell yield per femur during the 24 h after drug treatment (see Results). At 2 h the cell yield is indistinguishable from that of the controls, so surviving fractions are the same, expressed in either manner.

Drug doses were selected to give a drop in survival over one decade in unheated animals.

*Peripheral WBC counts.*—Groups of 8–10 mice were treated and blood samples were taken on Days 3, 5, 7, 10, etc., after treatment. Ten $\mu$l of blood was taken from the tail, into a heparinized capillary tube, and diluted in 20 ml of Isoton (Coulter Electronics Ltd). Heparinization was necessary because of a tendency for the blood of heated mice to clot very rapidly 3–5 days after treatment. Red blood cells were then lysed with Zaponin (Coulter Electronics Ltd) and white cells were counted in a Coulter Counter Model ZBI. Arithmetic means were taken for each group, and results were expressed as a percentage of the control counts on that day. This was because of the usual slight rise in WBC counts in control mice as a result of repeated bleedings.

Drug doses were chosen to match the stem-cell survival experiments where possible, but only lower doses of BCNU were compatible with survival for the length of the experiment.

### *Tumour*

The tumour used in this work was the RIF-1 tumour which has been previously described by Twentyman *et al.* (1980). It was grown i.m. in the leg by injection of $10^5$ cells from culture. Animals with tumours were treated when the tumours reached a size of 300–600 mm$^3$, usually Day 9–10 after inoculation, tumour volume being estimated from a calibration curve of tumour weight (proportional to tumour volume) as a function of the product of two mutually perpendicular leg diameters (Twentyman *et al.*, 1979). These leg diameters were measured with a specially made Perspex gauge. Tumour response was measured by two assays:

*Clonogenic tumour-cell survival.*—Tumours were excised 24 h after treatment, this being the earliest time at which most or all of the recovery from potentially lethal damage (PLD) is thought to be complete (Twentyman, 1977, 1979; Begg, 1980) yet it allows relatively little time for proliferation of surviving cells. Tumours were minced finely with scissors and disaggregated by 45 min stirred incubation in 1 mg/ml neutral protease (Sigma) in complete Eagle's minimal essential medium (MEM) supplemented with 20% newborn calf serum, as described by Twentyman & Yuhas (1980). The resulting single-cell suspension was counted with a haemacytometer, appropriate dilutions were made, and cells were plated in 90 mm Petri dishes in complete medium, as above. Dishes were incubated for 12–13 days in a gassing incubator and then the colonies were fixed, stained and counted. The plating efficiency (PE) for control tumours was 35–50%, and surviving fractions were calculated from the PE.

Drug doses were selected either to match the marrow survival experiments, or up to a dose calculated to give a clonogenic cell survival within the lower limit of assay, whichever was greater.

*Tumour growth delay.*—Groups of ~10 mice were used for each treatment and tumours were then measured twice weekly. Growth of each tumour was plotted, and the time taken for each to reach 4× the mean treatment volume was recorded. Arithmetic means for each group were calculated, and comparison with the control group allowed an estimate of the growth delay due to each treatment.

Drug doses were chosen to match the marrow-survival experiments as far as possible.

### RESULTS

### *Normal tissue*

*CFU-S.*—The drop in yield of nucleated cells per femur 24 h after drug treatment is shown in Fig. 1. For CTX, the drop appeared to be dose-dependent, with a 30% yield at 200 mg/kg in unheated animals, heated femur yields being consistently lower. For BCNU, the unheated yield was about 50% over the range of

FIG. 1.—Nucleated cells per femur 24 h after drug treatment. ●, unheated animals; ○, heated animals. Heat treatment was 45 min at 41°C. Each point shows the mean for 2–6 mice.

doses, again with yields from heated animals tending to be lower. Heat alone had no effect on cell yield at 24 h.

Dose–response curves obtained from unheated animals, both 2 and 24 h after CTX and BCNU, are shown in Fig. 2. Parameters describing these curves are given in Table I. The shoulder evident on the CTX curve at 2 h is eliminated by 24 h, and the slopes of the exponential parts are essentially the same. The BCNU curves are virtually identical at both assay times.

The effect of heat (45 min at 41°C) on these responses are shown in Fig. 3, and the parameters for these curves are also given in Table I. For CTX heat potentiates stem-cell damage at 2 h at doses only in excess of 100 mg/kg but when damage is assayed at 24 h, potentiation is evident at all drug doses. Again there is no evidence for a difference in slope in the exponential parts of the curves for heated animals. For BCNU heat potentiation of stem-cell killing is detected at both assay times, but at 2 h this is just a slight shift in the curve, with no significant change in slope, whilst at 24 h the effect is much more marked and there is a change in slope comparable to that seen for CTX at 24 h. Comparing slopes for heated and unheated response curves assayed at 24 h gives a Dose Modifying Factor (DMF)* of 2·0 for CTX and 2·1 for BCNU.

*Peripheral WBC counts.*—Results of two representative white-cell-count experiments are shown in Fig. 4. Separate panels show results for 200 and 100 mg/kg of CTX, respectively, in Expt 1 and for 30 mg/kg BCNU in Expt 2. Heat alone did not cause a major deviation from the control count at any time point in these or similar experiments. Both cytotoxic agents caused a drop in WBC count, with a nadir at Day 3. At 200 mg/kg CTX the effect of heat and drug was to decrease the nadir only slightly, but to maintain it until at least Day 5 and to delay recovery to normal levels by about 2 days. At 100 mg/kg the nadir was again only slightly depressed by the addition of heat, and the effect was almost to eliminate the Day 5 overshoot seen with CTX alone. The large error bars on Day 5 drug-treated points are because the recovery of WBC counts of mice in each group was not well synchronized. These results would seem to support the stem-cell survival data, in illustrating the heat potentiation of drug damage during the week after treatment.

* Defined as:

$$\frac{\text{Dose of drug to produce a given effect in control mice}}{\text{Dose of drug to produce the same effect in heated mice}}$$

FIG. 2.—Marrow stem-cell survival of unheated animals following CTX and BCNU. Assays were carried out 2 and 24 h after treatment. Each point represents the mean of 7–14 spleens. Lines fitted by least-squares regression.

TABLE I.—*Parameters describing stem-cell survival dose–response curves for unheated and heated animals treated with CTX or BCNU*

| | | Unheated | | Heated | |
|---|---|---|---|---|---|
| Drug | Assay time (h) | Slope ± 2 s.e. (kg/mg) | $D_0$ (mg/kg) | Slope ± 2 s.e. (kg/mg) | $D_0$ (mg/kg) |
| CTX | 2 | 0·011 ± 0·006 | 90·9 | 0·033 ± 0·009 | 30·3 |
| | 24 | 0·013 ± 0·002 | 76·9 | 0·026 ± 0·003 | 38·5 |
| BCNU | 2 | 0·045 ± 0·015 | 22·2 | 0·052 ± 0·006 | 19·2 |
| | 24 | 0·041 ± 0·006 | 24·4 | 0·085 ± 0·010 | 11·8 |

They particularly substantiate the 24h assay data, in that there is evidence for a potentiating effect of heat on drug damage at 100 mg/kg.

At 30 mg/kg BCNU, heat alone only slightly depressed the Day 3 nadir, but again delayed the recovery to normal levels by about 2 days, as with 200 mg/kg

FIG. 3.—The effect of heat (45 min at 41°C, open symbols) on marrow stem-cell survival after CTX and BCNU. Assays were carried out at 2 and 24 h after treatment. Each point represents the mean of 7–14 spleens. Lines fitted by least-squares regression. Closed symbols show values in untreated animals.

FIG. 4.—Changes with time in peripheral WBC count after treatment with heat alone (45 min at 41°C) (■— — —), drug alone (●——) or heat + drug (▲— —). Each point represents the mean of 7–10 animals and is expressed as a percentage of control count on the same day. Error bars show ± 2 s.e.

CTX. However, the nadir was not in this case maintained, perhaps because it reached only ~35%, compared with ~12% for 200 mg/kg CTX with heat. This result again corroborates the stem-cell survival assay measured at 24 h, in that at 30 mg/kg BCNU with heat, a delay in recovery to normal WBC levels, comparable with that with 100 mg/kg CTX and heat was obtained. It will be noted that the 24h stem-cell assay shows about the same amount of killing by 30 mg/kg BCNU and 100 mg/kg CTX, and also about the same degree of heat potentiation of this damage. It was not possible to get WBC counts for BCNU at 60 mg/kg to compare with CTX at 200 mg/kg, because of the toxicity of BCNU, particularly in combination with heat.

*Tumour response*

*Clonogenic tumour-cell survival.*—Results of two representative experiments are shown in Fig. 5. Parameters describing the exponential parts of the curves are given in Table II. Heat alone had no effect on tumour-cell survival. The RIF-1 tumour is sensitive to CTX, and cell killing is potentiated by heat (Fig. 5). However, the effect is to shift the curve downwards without altering its slope at doses above 75 mg/kg with heat. The DMF is thus dose-dependent, being at a maximum of 1·9 at 125 mg/kg, and gradually decreasing as the curves continue parallel. At 150 mg/kg the DMF is 1·6 and at 200 mg/kg is 1·4 (by extrapolation). Data from other experiments (not shown) indicated that the unheated curve continues straight while the heated one disappears below the limit of reliable assay.

In contrast, the RIF-1 tumour is resistant to BCNU, as shown in Fig. 5, and clonogenic cell killing is only slightly increased by heat. Again, the effects appears to be to shift the curve downwards at doses up to 45 mg/kg, but with greater potentiation at 60 mg/kg. These data yield DMFs varying from 2·0 for 30 mg/kg to 1·6 for 60 mg/kg (Table IV).

*Growth delay.*—Results of a tumour growth-delay experiment are given in Table III. It can be seen that again heat alone has no effect on tumour growth.

FIG. 5.—Clonogenic tumour cell survival after CTX or BCNU. ●, unheated animals; ○, heated animals. Heat treatment was 45 min at 41°C. Each point represents a tumour from 1 animal. Assays performed 24 h after treatment. Lines fitted by least-squares regression.

TABLE II.—*Parameters describing exponential parts of tumour-cell survival dose–response curves for unheated and heated animals treated with CTX or BCNU*

| | Unheated | | Heated | |
|---|---|---|---|---|
| Drug | Slope ± 2 s.e. (kg/mg) | $D_0$ (mg/kg) | Slope ± 2 s.e. (kg/mg) | $D_0$ (mg/kg) |
| CTX | 0·062 ± 0·006 | 16·1 | 0·059 ± 0·017 | 16·9 |
| BCNU | 0·028 ± 0·006 | 35·7 | 0·041 ± 0·010 | 24·4 |

Heat, however, substantially increases the growth delay caused by both 150 and 100 mg/kg CTX; the growth delay caused by 150 mg/kg CTX in unheated animals being the same as that caused by 100 mg/kg CTX in heated animals, which gives a DMF at 150 mg/kg of 1·5. This agrees well with the value of 1·6 at 150 mg/kg obtained from the clonogenic cell-survival assay.

RIF-1 is again seen to be refractory to BCNU treatment at the doses of 45 and 30 mg/kg. Although slightly longer growth delays are seen in heated groups, the differences are not significant at the 5% level.

Comparison of DMF values for normal and tumour tissues in these mice using the combination of heat with CTX or BCNU, can now be made to estimate a therapeutic ratio (TR) which may be defined as:

$$\frac{\text{DMF for tumour}}{\text{DMF for normal tissue}}$$

TABLE III.—*Tumour response of unheated and heated animals treated with CTX or BCNU, assayed by growth delay*

| Treatment | Time to reach 4 × treatment volume ± 2 s.e. (days) | Growth delay ± 2 s.e. (days) | *n* |
|---|---|---|---|
| Control | 4·8 ± 0·5 | | 10 |
| Heat alone | 5·1 ± 0·3 | 0·3 ± 0·6 | 10 |
| 150 mg/kg CTX | | | |
| Unheated | 19·8 ± 1·9 | 15·0 ± 2·0 | 11 |
| Heated | 27·0 ± 2·4 | 22·2 ± 2·5 | 7* |
| 100 mg/kg CTX | | | |
| Unheated | 13·3 ± 1·1 | 8·5 ± 1·2 | 10 |
| Heated | 20·1 ± 1·7 | 15·3 ± 1·7 | 10 |
| 45 mg/kg BCNU | | | |
| Unheated | 6·0 ± 0·7 | 1·2 ± 0·9 | 10 |
| Heated | 8·6 ± 2·6 | 3·8 ± 2·6 | 7* |
| 30 mg/kg BCNU | | | |
| Unheated | 5·6 ± 0·8 | 0·8 ± 0·9 | 10 |
| Heated | 6·8 ± 0·9 | 2·0 ± 1·0 | 10 |

* 3/10 died earlier.

TABLE IV.—*Effect of heat on tumour and marrow stem-cell survival at varying doses of CTX and BCNU, assayed 24 h after treatment*

| Drug | Dose (mg/kg) | $DMF_{tumour}$; | $DMF_{marrow}$ | TR* |
|---|---|---|---|---|
| CTX | 75 | 1·6 | 2·0 | 0·78 |
| | 125 | 1·9 | 2·0 | 0·91 |
| | 150 | 1·6 | 2·0 | 0·81 |
| | 200 | 1·4 | 2·0 | 0·69 |
| BCNU | 30 | 2·0 | 2·1 | 0·97 |
| | 45 | 1·7 | 2·1 | 0·81 |
| | 60 | 1·6 | 2·1 | 0·77 |

*TR < 1 implies therapeutic disadvantage.

(*i.e.* TR > 1·0 indicates a therapeutic advantage and TR < 1·0 indicates a therapeutic disadvantage). Values obtained by comparing DMFs obtained from the stem-cell survival data with those from clonogenic tumour-cell survival are presented in Table IV. For CTX the TR varies with dose, and it can be seen that the data indicate that there is no therapeutic gain from the combination of CTX with heat at 125 mg/kg, and at other doses there is a therapeutic loss. For BCNU a similar range of values is obtained, indicating no gain at 30 mg/kg and progressively greater therapeutic loss at doses up to 60 mg/kg.

### DISCUSSION

The elimination of the shoulder seen on the 2h stem-cell dose–response curve for unheated CTX-treated animals 24 h after treatment is in accordance with the observations of Hellman & Grate (1971), who assayed at 3 and 24 h after treatment in C3H/Hej mice. The effect of combining CTX with heat appears similar at both early and late assays, in that the shoulder on the 2h dose–response curve is maintained in heated animals (Fig. 3) and the slopes of the exponential parts of the 2h response curves do not differ from the slopes of the corresponding 24h curves (Table I). At 200 mg/kg there is about the same heat potentiation of damage (one decade) at both assay times.

For BCNU, the 2h stem-cell data for unheated animals presented in this paper agree closely with those of Wasserman *et al.* (1981) who quote a $D_0$ of 19 mg/kg, compared with our value of 22·2 mg/kg. Our data for both 2 and 24 h also agree with those of Van Putten *et al.* (1972) for 16 h after treatment with i.p. BCNU, but they also report a much steeper dose–response curve after s.c. administered drug. The s.c. route is obviously unsuitable for use in conjunction with an early assay, so all our observations were carried out using i.p. administration.

Despite the great similarity of the 2

and 24 h dose–response curves for unheated animals treated with BCNU (Table I, Fig. 2), the effect of heat in conjunction with BCNU differs markedly at the different assay times, and thus contrasts with the joint effect of heat and CTX. Assays at both 2 and 24 h after treatment show heat potentiation of BCNU damage at all dose levels, but 2 h after treatment there is only a slight downward shift of the curve, not altering its slope. However, at 24 h the effect is much greater, heat markedly increases the slope of the curve, and the potentiation at 60 mg/kg is by about 10-fold, compared with a factor of 2 for the 2h assay. It is not clear why heat potentiation of drug damage should be on a different time scale for different drugs, but evidently the balance of factors affecting the measurement of the stem-cell assay (detailed in the introduction) differs according to the exact nature of the heat potentiation, and from drug to drug.

The nadir of stem-cell survival after CTX has been shown to be at Day 1 after treatment by various workers, by both *in vivo* (Valeriote *et al.*, 1968) and *in vitro* assay (Brown & Carbone, 1971) and this would perhaps support the view that the 24h assay is the more useful. The fact that the peripheral WBC counts, being a measure of the end-product of stem cell killing, appear to agree more closely with the 24h stem-cell assay than with the 2h assay for both drugs (see Results) again argues that the 24h stem-cell assay data are the more relevant.

As already stated, 24 h after treatment is the optimum time for a reliable estimate of clonogenic tumour-cell survival, in that all or most repair of PLD is complete, but there has been little time for proliferation of clonogenic surviving cells. The agreement between the tumour-regrowth data and the clonogenic assay supports the validity of the 24h clonogenic assay. Hence the comparison of 24h stem-cell survival with 24h clonogenic tumour-cell survival is likely to give realistic estimates of TR for the combination of heat with the drugs tested. These estimates are most discouraging from a therapeutic viewpoint, as they indicate variation from no therapeutic gain to a considerable therapeutic loss, according to the dose of drug (Table IV). However, these studies only involve combinations where drug is given at the start of heating, and it is possible that the time scale of heat potentiation of drug toxicity may be substantially different for tumour and normal tissues, thus allowing a therapeutic advantage. Such an effect has been shown for the combination of waterbath heating and X-rays, using skin desquamation as the normal-tissue end-point (Hill & Denekamp, 1979).

It is interesting that very similar TRs were obtained for two drugs, CTX being very active against the RIF-1 tumour and BCNU relatively inactive against this tumour, even at very high doses. Despite the insensitivity of RIF-1 to BCNU, the toxicity observed was potentiated to about the same degree by the heat as was that of CTX, though the data are not as clear as those for CTX, being almost all within one decade of killing. This led to similar TRs, since the sensitivity of marrow stem cells was similar to both drugs over the dose ranges used. It would be interesting to investigate heat potentiation of the BCNU effect against a BCNU-sensitive tumour in the same strain of mouse, which we have not yet been able to do.

The lack of therapeutic advantage seen from this work is perhaps all the more discouraging from a clinical point of view because the temperature and time for which it is maintained (41°C for 45 min) constitute such a mild treatment. It is true that mice are not the ideal model animals for whole-body hyperthermia, being highly susceptible to heat stroke, but it seems reasonable to assume that, if animals do not succumb to heat stroke, the reactions of their haemopoietic systems might be typically mammalian. Much higher temperatures than 41°C are currently being tried for longer periods of local hyperthermia in clinical studies and, as already mentioned, it has been suggested that mild whole-body hyperthermia (at up to 41 °C) might be deliberately used in conjunction with local heating to reduce the problem of blood cooling. This problem of blood cooling remains despite the recent improvements in techniques of localizing administered heat. Our results suggest that any attempt to combine agents such as CTX or BCNU with such heating methods would be most unwise, and thus essentially agree with the findings of Rose *et al.* (1979). These workers used an incubator heating method, but unfortunately their heat doses (quoted as $>41{\cdot}5$°C for 20 min and $>41$°C for 30 min) which resulted from accumulation of body heat in an incubator at 38·7°C cannot be compared with ours. This is because the "temperature measurement" mice were anaesthetized with pentobarbitone, while the experimental mice were not. It is now known that pentobarbitone anaesthesia abolishes the animal's temperature-regulation mechanism, so the temperature of the monitored mice is unlikely to represent that of the experimental mice, and it is most likely that the unanaesthetized mice at a much lower temperature.

The work presented in this paper indicates that the simultaneous combination of heat and CTX or BCNU leads to no therapeutic gain, and under some conditions to therapeutic loss, the therapeutic ratios being estimated by comparing tumour toxicity and marrow toxicity.

We wish to thank Dr Peter Twentyman for many helpful discussions during the course of this work. We also thank Mrs Jill Shaw for caring for the animals used.

### REFERENCES

Begg, A. C., Fu, K. K., Kane, L. J. & Phillips, T. L. (1980) Single-agent chemotherapy of a solid murine tumour assayed by growth delay and cell survival. *Cancer Res.*, **40**, 145.

Brown, C. H. III., & Carbone, P. P. (1971) Effects of chemotherapeutic agents in normal mouse bone marrow grown *in vitro*, *Cancer Res.*, **31**, 185.

Chrisman, C. L. & Baumgartner, A. P. (1980) Micronuclei in bone marrow cells of mice subjected to hyperthermia. *Mutat. Res.*, **77**, 95.

Hahn, G. M. (1978) Interactions of drugs and hyperthermia *in vitro* and *in vivo*. In *Cancer Therapy by Hyperthermia and Radiation* (Ed. Streffer). Baltimore: Urban & Schwarzenberg. p. 72.

Hellman, S. & Grate, H. E. (1971) Effect of cyclophosphamide on the murine haematopoietic stem cell compartment as measured by different assay techniques. *Blood*, **38**, 706.

Hill, S. A. & Denekamp, J. (1979) The response of six mouse tumours to combined heat and X-rays: Implications for therapy. *Br. J. Radiol.*, **52**, 209.

Honess, D. J. & Bleehen, N. M. (1980) Effects of the combination of hyperthermia and cytotoxic drugs on the skin of the mouse foot. In *Proc. 1st Meeting European Group of Hypertherima and Radiation Oncology* (Ed. Archangeli & Mauro). New York: Masson. p. 151.

Hume, S. P., Robinson, J. E. & Hand, J. W. (1979) The influence of blood flow on temperature distribution in the exteriorised mouse intestine during treatment by hyperthermia. *Br. J. Radiol.*, **52**, 219.

Marmor, J. B. (1979) Interactions of hyperthermia and chemotherapy in animals. *Cancer Res.*, **39**, 2269.

Overgaard, J. (1976) Combined Adriamycin and hyperthermia treatment of a mammary carcinoma. *Cancer Res.*, **36**, 3077.

Rose, W. C., Veras, G. H., Laster, W. R. Jr., & Schabel, F. M., Jr (1979) Evaluation of whole body hyperthermia as an adjunct to chemotherapy in murine tumours. *Cancer Treat. Rep.*, **63**, 1311.

Sabio, H., Elkon, D. & Baker, D. (1981) Effect of hyperthermia on murine myeloid proliferation. *Proc. Am. Assoc. Cancer Res.*, **22**, 238.

Symonds, R. P., Wheldon, T. E., Clarke, B. & Bailey, G. (1981) A comparison of the response to hyperthermia of murine haemopoietic stem cells (CFU-S) and L1210 leukaemia cells: Enhanced cell killing of leukaemic cells in presence of normal marrow cells. *Br. J. Cancer*, **44**, 682.

Till, J. E. & McCulloch, E. A. (1961) A direct measurement of the radiation sensitivity of normal mouse bone marrow cells. *Radiat. Res.*, **14**, 213.

Twentyman, P. R. (1977) The sensitivity to cytotoxic agents of the EMT6 tumour *in vivo*: Comparison of data obtained using tumour volume measurement and *in vitro* plating. I. Cyclophosphamide. *Br. J. Cancer*, **35**, 208.

Twentyman, P. R. (1979) Timing of assays: An important consideration in the determination of clonogenic cell survival both *in vivo* and *in vitro*. *Int. J. Radiat. Oncol. Biol. Phys.*, **5**, 1213.

Twentyman, P. R., Brown, J. M., Gray, J. W., Franko, A. J., Scoles, M. A. & Kallman, R. F. (1980) A new mouse model tumour system (RIF-1) for comparison of endpoint studies. *J. Natl Cancer Inst.*, **64**, 595.

Twentyman, P. R., Kallman, R. F. & Brown, J. M. (1979) The effect of time between X-irradiation and chemotherapy on the growth of three solid mouse tumours. I. Adriamycin. *Int. J. Radiat. Oncol. Biol. Phys.*, **5**, 1255.

Twentyman, P. R., Morgan, J. E. & Donaldson, J. (1978) Enhancement by hyperthermia of the effect of BCNU against the EMT6 mouse tumour. *Cancer Treat. Rep.*, **62**, 439.

Twentyman, P. R. & Yuhas, J. M. (1980) Use of a bacterial neutral protease for disaggregation of mouse tumours and multicellular tumour spheroids. *Cancer Letters*, **9**, 225.

VALERIOTE, F. A., COLLINS, D. C. & BRUCE, W. R. (1968) Haematological recovery in the mouse following single doses of gamma radiation and cyclophosphamide. *Radiat. Res.*, **33**, 501.

VAN PUTTEN, L. M., LELIEVELD, P. & KRAM-IDSENGA, L. K. J. (1972) Cell cycle specificity and therapeutic effectiveness of cytostatic agents. *Cancer Chemother. Rep.*, **56**, 691.

WASSERMAN, T. H., PHILLIPS, T. L., ROSS, G. & KANE, L. J. (1981) Differential protection against cytotoxic chemotherapeutic effects on bone marrow CFUs by WR 2721. *Cancer Clin. Trials*, **4**, 3.